# Comparative metabolomics and glycolysis enzyme profiling of embryogenic and nonembryogenic grape cells

**DOI:** 10.1002/2211-5463.12415

**Published:** 2018-04-17

**Authors:** Jonathan Parrilla, Cécile Gaillard, Jérémy Verbeke, Mickaël Maucourt, Radoslav A. Aleksandrov, Florence Thibault, Pierrette Fleurat‐Lessard, Yves Gibon, Dominique Rolin, Rossitza Atanassova

**Affiliations:** ^1^ Laboratoire EBI‐ Ecologie et Biologie des Interactions Équipe SEVE‐Sucres et Échanges Végétaux‐Environnement UMR 7267 Centre National de la Recherche Scientifique Université de Poitiers France; ^2^ GReD. UMR CNRS 6293 ‐ INSERM U1103 Université Clermont‐Auvergne CRBC Faculté de médecine Clermont‐Ferrand France; ^3^ Laboratoire Biologie du Fruit et Pathologie UMR 1332 Institut National de la Recherche Agronomique Université de Bordeaux Villenave d'Ornon France; ^4^ Plateforme Métabolome du Centre de Génomique Fonctionnelle Bordeaux MetaboHUB Institut National de la Recherche Agronomique Villenave d'Ornon France; ^5^ Institute of Molecular Biology Bulgarian Academy of Sciences Acad Sofia Bulgaria

**Keywords:** cell proliferation and cell fate, glycolysis enzyme activities, grape embryogenic and nonembryogenic cells, metabolic behavior

## Abstract

A novel biological model was created for the comparison of grapevine embryogenic cells (EC) and nonembryogenic cells (NEC) sharing a common genetic background but distinct phenotypes, when cultured on their respective most appropriate media. Cytological characterization, ^1^H‐NMR analysis of intracellular metabolites, and glycolytic enzyme activities provided evidence for the marked metabolic differences between EC and NEC. The EC were characterized by a moderate and organized cell proliferation, coupled with a low flux through glycolysis, high capacity of phosphoenolpyruvate carboxylase and glucokinase, and high oxygen consumption. The NEC displayed strong anarchic growth, and their high rate of glycolysis due to the low energetic efficiency of the fermentative metabolism is confirmed by increased enolase capacity and low oxygen consumption.

**Abbreviations**
EC: embryogenic cells
NEC: nonembryogenic cells
PCA: principal component analysis
PCV: packed cell volume
TEM: transmission electron microscopy

Many plant species are able to produce new embryos from single somatic cells as a pertinent strategy of asexual reproduction 1. Such embryogenic cells are a powerful tool for micropropagation and large‐scale *in vitro* multiplication of species with limited seed production, germplasm conservation, as well as for genetic transformation of many woody plants, for example, grapevine. To better understand somatic embryogenesis, it is also important to keep in mind that the zygote is a free cell, whose fate is independent of any surrounding tissue, avoiding neighboring cell communications, and evolving under endosperm nutritional and hormonal signaling. The fact that functional embryos can develop from somatic cells demonstrates that the genetic program for embryogenesis is totally confined within the cell and can function completely in the absence of gene products from the maternal environment 2, 3. The similarity between zygotic and somatic embryogenesis is striking and remarkable, considering that somatic embryos develop completely outside of both the physical constraints and the informational context of maternal tissue 4, 5. However, unlike zygotic embryos, the somatic embryos developing *in vitro* need neither desiccation nor dormancy 6, 7.

Embryogenic cell (EC) cultures are suspensions of pro‐embryogenic masses, which consist of aggregates of small cells 8, 9, 10, 11, 12. Interestingly, the formation of cellular groups is thought to be a prerequisite for maintaining their ability to differentiate into somatic embryos, thereby allowing plant regeneration 13. Auxins are necessary for preserving the embryogenic character of cells, but they inhibit the induction of somatic embryogenesis 14. Inversely, the nonembryogenic cells (NEC) do not form embryos in culture, but continue to proliferate as nonorganized cells 15. Although metabolic studies have been performed on embryogenic and nonembryogenic callus tissue from sugar cane 16, 17, the comparisons at cellular level of EC and NEC still remain limited, either to hormonal level 18, 19, 20, 21, or to starch synthesis 15 and cell wall components 12.

We performed an in‐depth comparison of grape EC and NEC sharing a common genetic background, but cultured on particular nutritional media for maintaining their specific cell fate determination. The comparative analysis of cytological characteristics, growth kinetics, cellular metabolites, and glycolysis enzymes of grape EC and NEC revealed marked differences in their proliferation, metabolic behavior, and glycolytic metabolism.

## Materials and methods

### Plant material

Two grapevine cell suspensions (EC and NEC), both derived from the rootstock hybrid 41B, the most commonly used rootstock in the vineyards of Champagne (a hybrid between *Vitis vinifera* L. cv. Chasselas x *Vitis berlandieri* P.), were used. The EC suspension was subcultured every 2 weeks by transferring 0.3 mL of packed cell volume (PCV) into 25 mL of a half‐strength Murashige and Skoog medium, containing glycerol (4.6 g·L^−1^) and maltose (18 g·L^−1^) as carbon sources, as well as naphthoxyacetic acid (NOA) (1 mg·L^−1^) and acid hydrolysate of casein. The NEC suspension was initiated using stem fragments of *in vitro* plants regenerated from the EC and, after its establishment, was subcultured every 2 weeks by transferring 1 mL PCV in 25 mL of fresh Gamborg medium complemented with sucrose (20 g·L^−1^), 1‐naphthalene‐acetic acid (NAA) (0.18 mg·L^−1^), and 6‐benzylaminopurine (BAP) (1 mg·L^−1^). Both cell suspensions grew under the same controlled physical conditions: constant shaking (110 r.p.m.), in darkness, and at 21 °C.

### Microscopy

The samples were fixed in 2% paraformaldehyde/0.5% glutaraldehyde with 0.1M Sörensen buffer pH 7.3, for 45 min, at 24 °C. After washing in the same buffer supplemented with 7.5% sucrose, the postfixation in 1% osmium tetroxide was performed for 5 min. The dehydrated samples were embedded in London Resin White and incubated 24 h at 60 °C for polymerization. Sections were obtained using EMUC6 Leica Microtome. Periodic acid/Schiff (PAS) reaction was used for starch visualization on semi‐thin sections (500 nm) fixed on polylysinated (1 mg·mL^−1^; w/v) slides. For transmission electron microscopy (TEM) on Jeol JEM 1010 microscope at 80 kV, the ultra‐thin sections (60 nm) were collected on gold grids and stained with uranyl acetate and lead citrate.

### Growth kinetics and cyclin gene expression

Cells were filtered, weighted for growth curves, and frozen in liquid nitrogen every 2 days within the 2 weeks time of culture. RNA was extracted using the Spectrum™ Plant Total RNA Kit (SIGMA‐Aldrich, Saint‐Quentin Fallavier, France) and retrotranscribed with M‐MLV retrotranscriptase (Promega, Charbonnières‐les Bains, France) following the respective manufacturers’ instructions. Cyclins’ expression was then determined by qRT/PCR (Promega qPCR Master Mix, Mastercycler realplex^2^). Four housekeeping genes have initially been tested: two elongation factors (VvEF1a, VvEF1 g), actin (VvACT1) and glyceraldehyde‐3‐phosphate dehydrogenase (VvGAPDH). Of all these, actin was the most stable and therefore chosen as a reference in our cellular models. The cyclin expression was normalized toward the actin as reference gene, using the 2^−ΔCt^ method and the following respective primers:

*VvCycA2;2* (XM_010650250.1)

F 5′‐CATGTTGCCAGGTCGATGTAAC‐3′; R 5′‐GAATGGCTCTGACATCATACAAC‐3′

*VvCycD3;3* (XM_002285284.3)

F 5′‐GGCTGGCATTTCCGAACAGAAAGG‐3′; R 5′‐GGGAACTGGGAACTGGGAAGAGAC‐3′

*VvActin* (XM_002282480.3)

F (5′‐GCATCCCTCAGCACCTTCCA‐3′; R 5′‐AACCCCACCTCAACACATCTCC‐3′.

### Metabolomic analysis

For the ^1^H‐NMR analysis, polar metabolites were extracted, titrated, lyophilized (EZ Dry‐FTS system), solubilized, and pretreated as described by Moing *et al*. 22. ^1^H‐NMR spectra were recorded at 500.16 MHz and 300K on a Bruker Avance III spectrometer using a 5 mm inverse probe and an electronic reference for quantification (Digital ERETIC, Bruker TopSpin 3.0). The assignments of metabolites in the NMR spectra were made by comparing the proton chemical shifts with literature values 23, 24, 25, with the spectra of authentic compounds recorded under the same buffer conditions and by spiking the samples with standards. Metabolite concentrations in the NMR tube were calculated using Analytical Profiler mode of AMIX software (version 3.9.10, Bruker) for calculation of resonance areas, followed by data export to Excel software. 2D‐homonuclear correlation spectroscopy (^1^H‐^1^H COSY) experiments were carried out to verify the identity of known compounds and to check whether unknown signals really correspond to different compounds. Starch was determined as described in Hendriks *et al*. 26.

### Oxygen consumption

The consumption of oxygen (O_2_) by both types of cells is measured using the Clark electrode (DUAL DIGITAL MODEL 20, RANK BROTHERS LTD). The rate of oxygen consumption was followed during 20 min on 20 mg cells maintained in the dark and was estimated in nmols O_2_·min^−1^·g^−1^ dry weight 27.

### Enzyme activities

Extraction was performed as described by Nunes‐Nesi *et al*. 28. All enzyme activities were carried out on a robotized platform 29 and assayed as described, respectively: phosphoglucose isomerase—PGI 30; enolase and triose‐phosphate isomerase—TPI 31; phosphoglucomutase—PGM 32; pyruvate kinase—PK, glucokinase—GK, fructokinase—FK, phosphoenolpyruvate carboxylase—PEPC and ATP‐dependent phosphofructokinase—PFK 29; phosphoglycerokinase—PGK 33; aldolase 34.

## Results

### Cytological characterization

**Figure 1:**
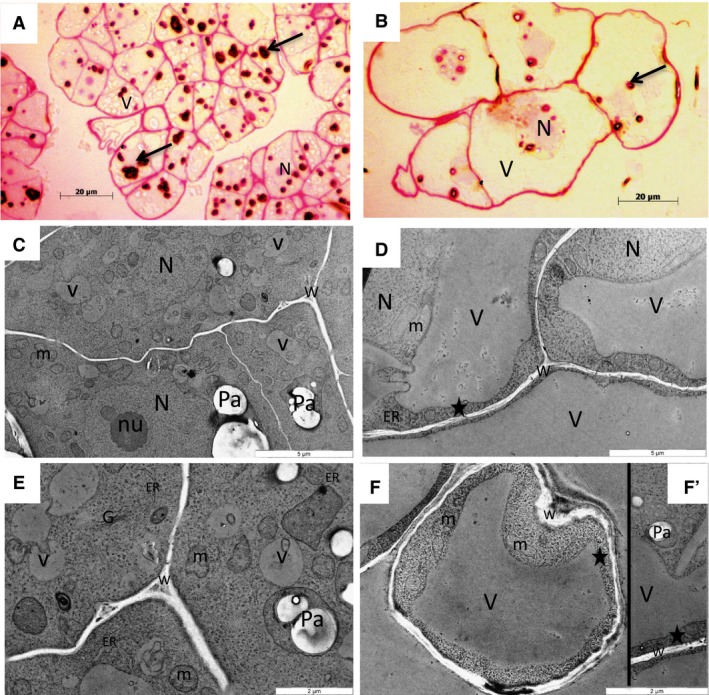
Microscopy observation of grape EC and NEC. Photonic microscopy: A. Clusters of numerous and small‐sized EC, with the nuclei (N) in the central part, small vacuoles (v) and many amyloplasts (arrow); B. small groups of large NEC with the nuclei at the cell periphery, one or two large vacuoles (V) and some amyloplasts (arrow). Transmission microscopy: C and E. In EC: many small vacuoles (v) in the dense cytoplasm, large starch grains in amyloplasts (Pa), many mitochondria (m), long profiles of endoplasmic reticulum (er), and Golgi stacks (G); D, F, and F’. In NEC: thin layer of cytoplasm (asterisk) adjacent to the cell wall (W), long mitochondria profiles, amyloplasts with small starch grains, short profiles of endoplasmic reticulum PFA/glutaraldehyde/OsO_4_/LRW. A and B. PAS staining—starch grains were purple‐stained and cell walls pink‐stained; C‐F’. uranyl/lead contrast—starch grains were white‐stained because of their high optic density.

The cytological study of both cell types harvested during the growth phase revealed important differences (Fig. 1). The EC clusters displayed an apparent homogeneity mimicking sand grains, composed of distinct groups of many small cells (10 to 20 μm wide) surrounded by a kind of thin gel coat (Fig. 1A). Inversely, the NEC were larger in size (50 to 60 μm in size) and formed very small groups of several adjacent cells (Fig. 1B). The nucleo‐cytoplasmic ratio of EC was significantly higher (0.60 ± 0.09) compared to that of the NEC (0.44 ± 0.10). The nuclei of EC were localized in the cell center, while the nuclei of NEC were usually at the cell periphery. The vacuolar apparatus of EC was composed of several small vacuoles (1 to 2 μm) embedded in a dense cytoplasmic matrix (Fig. 1C,E). The NEC presented one or two large vacuole(s), reaching 20 to 40 μm, compressing the cytoplasm as a thin layer to the cell wall (Fig. 1D,F). The EC presented a lot of large starch grains in amyloplasts 1 to 3 μm wide (mean 2.26 ± 0.94) (Fig. 1A,C, and E), while the NEC contained smaller amyloplasts (mean 1.17 ± 0.26) without any important starch reserves (Fig. 1B,D,F, and F’). In EC, abundant and small‐sized mitochondria (0.5 to 1 μm in width) with dense matrix and large cristae occurred together with many ribosomes forming polysomes, Golgi stacks, and a large amount of long endoplasmic reticulum profiles of rough‐type. By contrast, NEC mitochondria were often elongated (2 μm long and 1 μm large) with large electron lucent cristae and the endoplasmic reticulum had very short profiles.

### Proliferation characteristics

**Figure 2:**
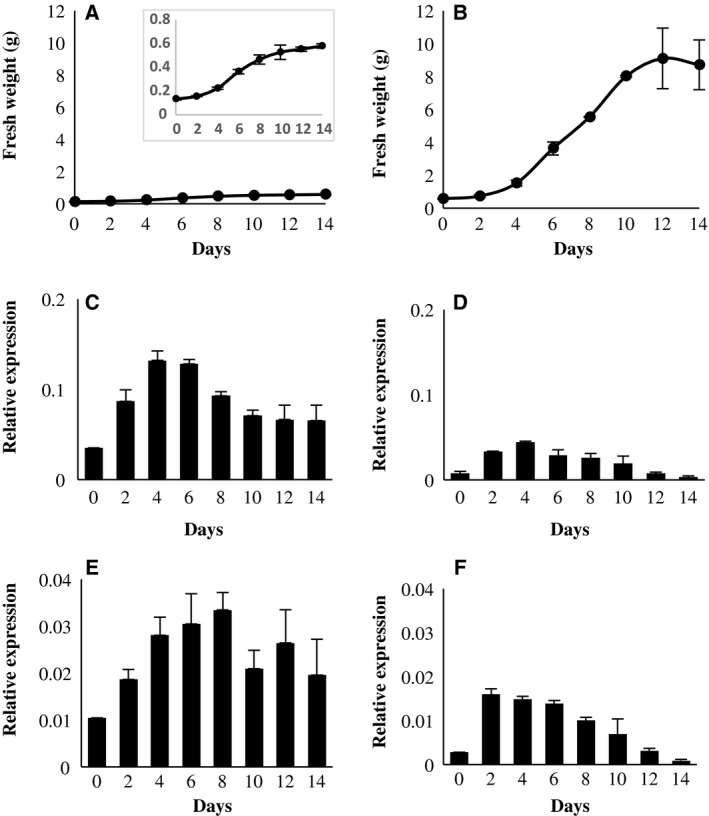
Growth curves of grape EC (A) and NEC (B) obtained using the fresh weight of filtered cells at each time point during the culture period (mean values from three independent biological replicates). Expression profiles of *CycD3;3* (C and D) and *CycA2;2* (E and F) genes, respectively, in EC (C and E) and NEC (D and F) (mean values from three biological replicates).

The different proliferation behavior of both cell types was reflected by their distinct growth curves, obtained through the fresh mass (Fig. 2A,B). The EC were characterized by slow and progressive proliferation, revealing their aptitude to grow for at least 1 month on the same culture medium without an apparent stationary phase (Fig. 2A). By contrast, the NEC displayed three phases: latent (from 0 to 2 day), growth (from 2 to 8 day), and stationary (from 10 to 14 day), and their survival was strongly dependent on subculture in a fresh nutritional medium (Fig. 2B).

In this regard, the RT/qPCR analysis of two genes encoding cyclins *VvCycD3;3* and *VvCycA2;2* surprisingly demonstrated a higher level of expression in EC (Fig. 2C,D) compared to that of NEC (Fig. 2E,F). At day 2 after subculture, EC presented a twofold initial increase in cyclin gene expression that reached a maximum at days 4 and 6 and at days 6 and 8, respectively, for *VvCycD3;3* and *VvCycA2;2*. After day 8, their expression remained stable at a level similar to that at day 4. An at least threefold more important expression of *VvCycD3;3* was observed, when compared to that of *VvCycA2;2*. Conversely, NEC demonstrated a lower level and a transient pattern of expression induction for both cyclins. At day 2, they displayed an initial fivefold to sixfold induction, respectively, for *VvCycD3;3* and *VvCycA2;2*, with a maximum at day 4, followed by a relatively stable level during the growth phase, and a critical drop before to reach the initial expression level at day 14.

### Comparison of the intracellular metabolites

The intracellular metabolite comparison of the EC and NEC was achieved by quantitative proton NMR analysis at seven time points during the 14 days of culture. We quantified six soluble sugars (glucose, fructose, maltose, sucrose, galactose, and fucose), two alcohol sugars (glycerol and inositol), seven organic acids (acetate, fumarate, citrate, succinate, formate, galacturonate, and malate), 15 amino acids (alanine, asparagine, aspartate, gamma amino butyric acid, glutamine, glutamate, isoleucine, leucine, histidine, phenylalanine, serine, tryptophan, valine, proline, and tyrosine), and two polyamines (cadaverine and putrescine) in the two cell types. In total, ^1^H NMR enabled the detection of 42 metabolites, from which 36 could be identified. This result was in line with the previously described detection capacity of the chosen method 35.

**Figure 3:**
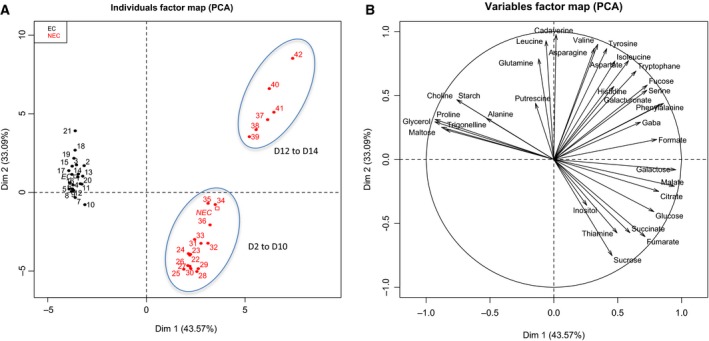
^1^H‐NMR analysis of intracellular soluble metabolites (sugars, amino acids, organic acids, etc.) throughout the growth kinetics of both cell cultures. Principal component analysis (PCA) by individual factor map (A) and variable factor map (B) of EC (black) and NEC (red). The numbers correspond to a total of 42 samples (three biological replicates for the seven time points of harvest during fourteen days of subculture for each of both cell types). Starch content was enzymatically determined.

Principal component analysis (PCA) was used to visualize the discriminating differences between both cell types (Fig. 3A). The first PCA axis, corresponding to 42.02% of the variance, revealed the spectacular cleavage between EC and NEC. The EC were perfectly clustered throughout the subculture period, displaying a high level of homogeneity. They were characterized by low levels of both maltose and glucose and by high levels of glycerol, alanine, proline, and some compounds related to the B group vitamins—choline and trigonelline, as detailed in Fig. 3B.

The NEC displayed two internal groups reflecting a changing metabolite profile during the subculture period (Fig. 3A,B). The evidence for this particularity was provided by the second PCA axis responsible for 32.72% of the variance (Fig. 3A). The first group of NEC corresponded to the latent and growth phases from day 0 to day 10, while the second corresponded to the establishment of the stationary phase (days 12 and 14). The NEC in proliferation presented high concentrations of soluble sugars (sucrose and glucose) and organic acids (citrate, fumarate, succinate, malate) (Fig. 3B).

The second axis was positively correlated with the intracellular concentration of some amino acids (aspartate, valine, asparagine, leucine, tryptophan, histidine, and phenylalanine) and the polyamine cadaverine. The concentration of free amino acids sharply increased at days 12 and 14 in the NEC, suggesting an induction of proteolysis at the stationary phase. The appearance of fucose, a deoxyhexose largely involved as the terminal sugar of protein N‐glycosylation, also confirmed this possibility. Another fact arguing in favor of the enhanced cell death at this latter phase concerned the accumulation of galacturonic acid and fucose, as valuable indicators of cell wall degradation.

**Figure 4:**
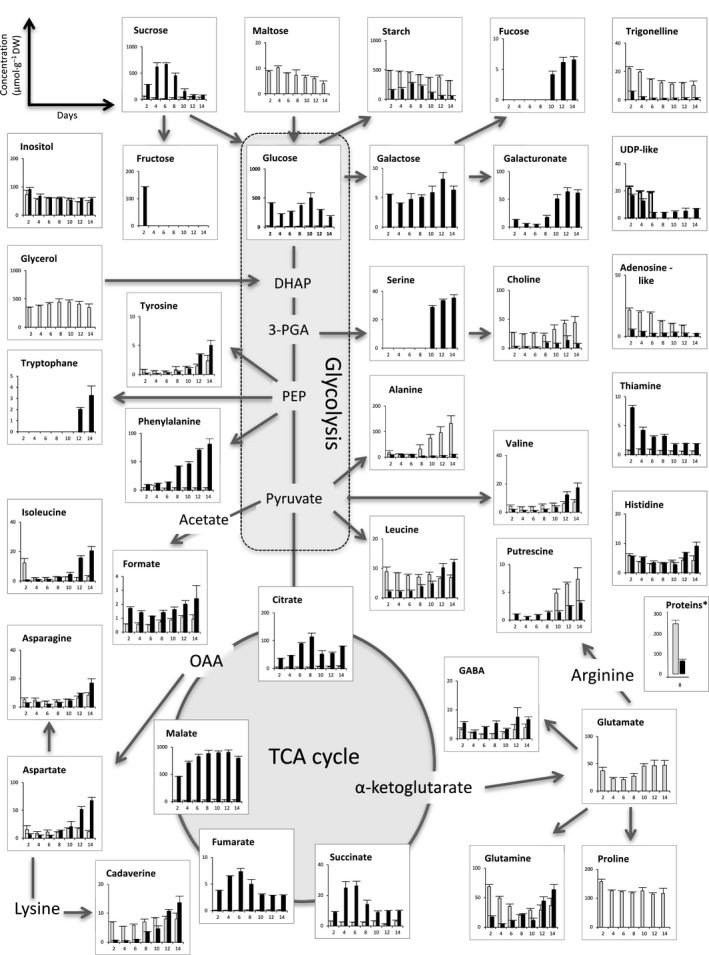
Quantitative comparison of the intracellular metabolites of both cell types related to glycolysis pathway and TCA cycle (mean values from three biological replicates): EC (columns in gray) and NEC (columns in black). Starch content was enzymatically determined. *Total proteins (mg·g^−1^ dry weight) were measured at day 8.

A quantitative comparison of identified cellular metabolites, performed by ^1^H‐NMR analysis of both cell types during 14 days of the subculture period, is presented in detail in Fig. 4 and S1. Despite the relatively similar concentrations of sucrose and glucose in the NEC, fructose was only detected at day 2 and its level was approximately one‐third that of glucose. Throughout this subculture period, starch concentration in the EC was higher than that in the NEC. In the EC, the starch content remained stable during 14 days, while in the NEC, it dropped down at the stationary phase. Two other compounds considered as compatible osmolytes, the amino acid proline and the amino alcohol choline (a quaternary ammonium), displayed relatively stable concentrations all over the proliferation of EC, but they were almost undetectable in the NEC. The total protein content of EC at day 8 was four times higher than these of NEC. The unique metabolite presenting the same very constant concentration for both cell types was inositol, an important source of key second messengers for the intracellular hormonal signaling.

### Comparison of culture medium metabolites

**Figure 5:**
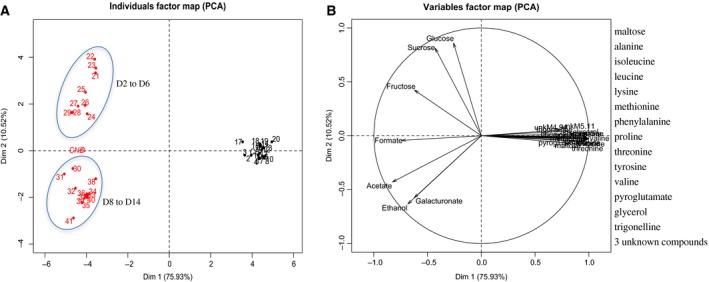
^1^H NMR analysis of the soluble metabolites of both cell culture media (sugars, amino acids, organic acids, etc.) throughout the growth kinetics of both cell cultures. Principal component analysis (PCA) by individual factor map (A) and variable factor map (B) of EC (black) and NEC (red). The numbers correspond to a total of 42 samples (three biological replicates for the seven time points of harvest during fourteen days of subculture, for each of both cell types).

In complement to the intracellular metabolites, a metabolic profiling ^1^H NMR study of the culture media was carried out. PCA was performed using all compiled data that were obtained throughout cell proliferation. As shown in Fig. 5, the first axis displays 75.93% of variance, which highlights the strong discrimination of the media used to culture EC and NEC. As both cell types were cultured on two distinct nutritional media, the comparison of extracellular metabolites allowed a better understanding of their nutritional behavior. Once again, the metabolites of the EC medium were perfectly matching and form a unique homogenous group (Fig. 5A). As expected, the culture medium of EC was characterized by high content of maltose, glycerol, and amino acids during the growth phase (Fig. 5B). Inversely, the metabolites of the NEC medium manifested two separate groups, corresponding, respectively, to the first part of the growing curve (days 0‐6) and to the second part (days 8‐14) (Fig. 5A). Elevated concentrations of sucrose, glucose, and fructose predominated between day 0 and day 6, while acetate, ethanol, and glucuronate accumulated mainly between days 8 and 14 (Fig. 5B).

**Figure 6:**
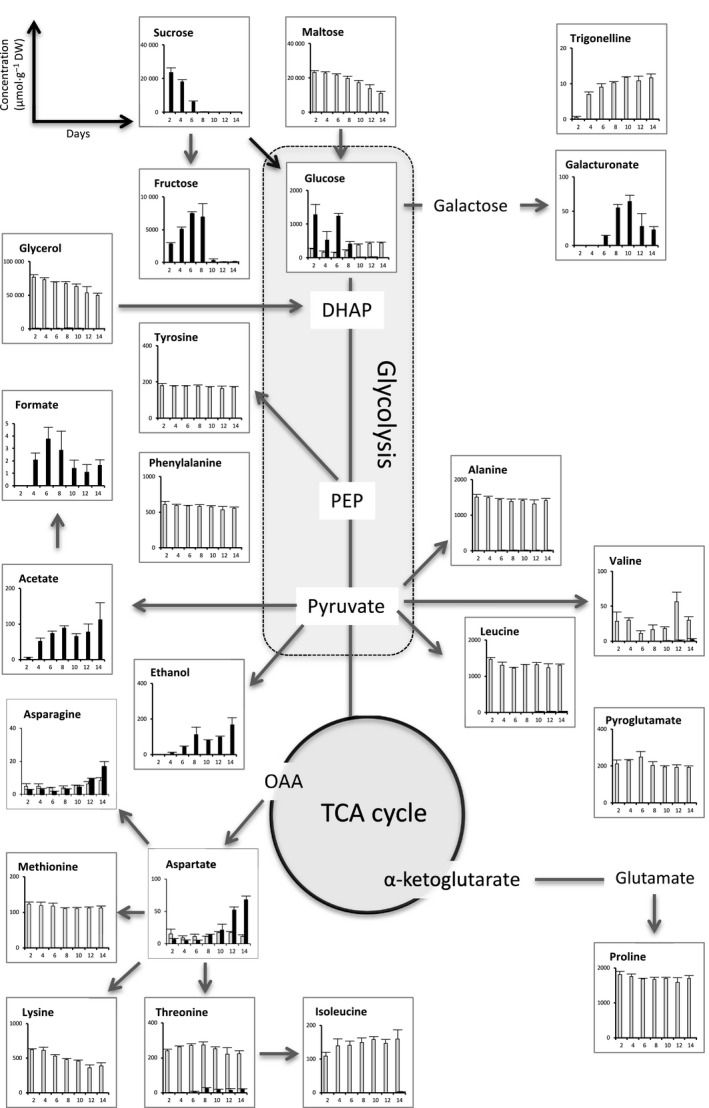
Quantitative comparison of the metabolites in culture medium of each cell type related to glycolysis pathway and TCA cycle (mean values from three biological replicates): EC (columns in gray) and NEC (columns in black).

The detailed analysis of all detected compounds in the culture media was presented in Figs 6 and S2. In EC, the carbon sources maltose and glycerol were used with parsimony throughout the 14‐day period, as additionally confirmed by the very low and relatively stable level of glucose. A second specific characteristic consisted in the maintaining of a very stable level of amino acids concentration during the subculture period, apparently due to the presence of casein hydrolysate, but also revealing a relatively low consumption by EC.

In the culture medium of NEC, the three soluble sugars (i.e., sucrose and its cleavage products glucose and fructose) were detected. Sucrose concentration displayed a rapid early decline, during the cell growth phase, and a complete depletion at day 8. This phenomenon was possibly due to loosely bound parietal invertases, which efficiently hydrolyze sucrose in culture medium. Between the two products of sucrose cleavage, glucose appeared as the preferred substrate of hexose transporters and sugar metabolism, as it was undetectable from day 8, while fructose was still measurable at day 10. The fact that ethanol, acetate, and formic acid accumulated over time in the culture medium revealed that anaerobic metabolism (fermentation) was taking place in these cells.

### Oxygen content as evidence for cell metabolic activity

**Figure 7:**
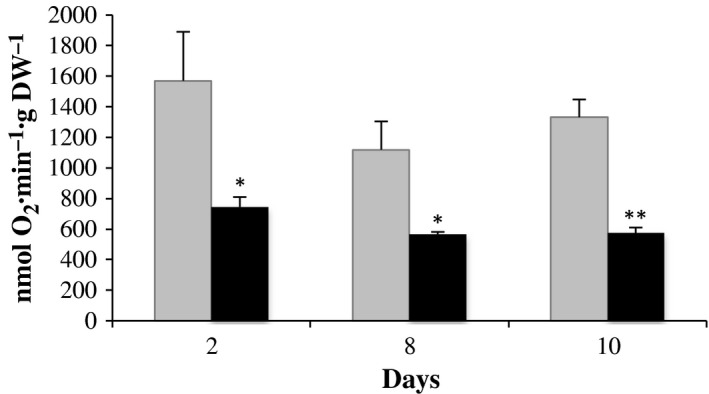
Rate of oxygen consumption throughout cell growth: day 2 (latent phase), day 8 (growth phase), and day 10 (stationary phase); EC (columns in gray) and NEC (columns in black). The results of Kruskal–Wallis test are presented as follows: *P value < 0.05; **P value < 0.01.

To obtain an appropriate estimation for the level of the oxidative phosphorylation in EC and NEC, oxygen consumption was measured with an oxygen electrode. The chosen time points corresponded to each of the three phases of proliferation, that is, latent (days 0‐2), growth (days 2‐8), and stationary (days 10‐14). The oxygen content was normalized to the dry weight of cells, thus minimizing the cell size differences. Despite a certain decline of the oxygen level in EC at day 8, the rate of electron flow to oxygen remained relatively stable for both cells types and especially for the NEC. The most striking observation consisted in the fact that the EC consumed twice as much oxygen as the NEC (Fig. 7).

### Profiling of enzyme activities in the glycolysis pathway

**Figure 8:**
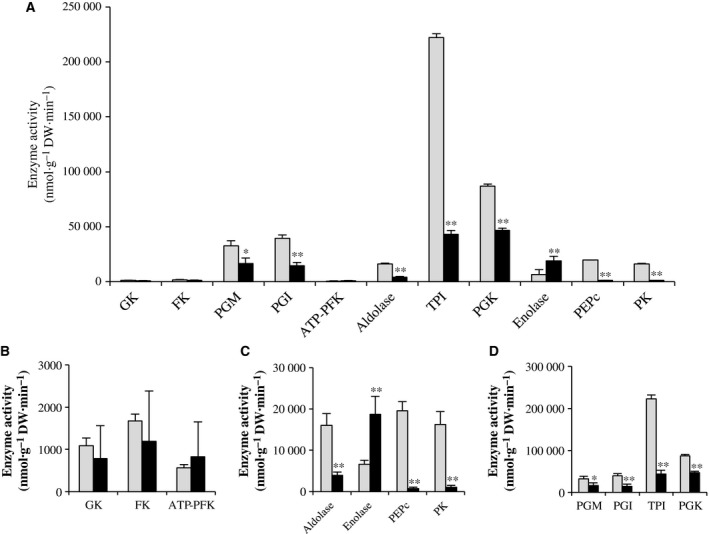
Activity of the glycolysis pathway enzymes: GK—glucokinase, FK—fructokinase, PGM—phosphoglucomutase, PGI—phosphoglucose isomerase, PFK—phosphofructokinase, aldolase, TPI—triose‐phosphate isomerase, PGK—phosphoglycerokinase, enolase, PEPC—phosphoenolpyruvate carboxylase, PK—pyruvate kinase. A. The activity of all studied enzymes is presented at the same scale. For a better visualization, B, C, and D present the maximum capacity of each from three groups of enzymes with comparable levels: EC (columns in gray) and NEC (columns in black). The results of Kruskal–Wallis test are presented as follows: *P value < 0.05; **P value < 0.01.

The profiles of the capacities (i.e., activities measured under substrate saturating conditions) of the glycolysis enzymes measured at day 8 were very different between the two types of cells (Fig. 8A,B,C,D). The twelve measured enzymes could be clustered in three categories based on their activities, which ranged from 2000 to 200 000 nmol·g^−1^ DW·min^−1^. Both cell types were characterized by enzymes of the irreversible steps of glucose metabolism (glucokinase, fructokinase, phosphofructokinase) with high capacities (Fig. 8B). The EC displayed higher capacities of enzymes catalyzing the reversible reactions in the upper part of glycolysis (phosphoglucose isomerase, aldolase, triose‐phosphate isomerase, phosphoglucomutase, phosphoglycerokinase) as well as phosphoenolpyruvate carboxylase and pyruvate kinase, which are both situated at the lower part of glycolysis and use phosphoenolpyruvate as substrate (Fig. 8C,D). Inversely, the NEC were characterized by an important enolase activity and by very low phosphoenolpyruvate carboxylase and pyruvate kinase activities (Fig. 8C).

## Discussion

The main challenge of the present study was to compare the metabolic behavior of EC and NEC sharing a common genotype but evolving as two independent phenotypes maintained on their most appropriate culture media. It should be stressed that none of the cell types can maintain its phenotype and specific cell fate determination in the opposite medium.

### Cytological characteristics and proliferation specificities

The EC characteristics, dense cytoplasm, small vacuoles, high nucleo‐cytoplasmic ratio, numerous polysomes, active mitochondria, ER, and Golgi (Fig. 1A,C, and E) argue in favor of a cell division coupled with cytoplasmic growth, which implies the accumulation of macromolecules and cellular components, as described for meristematic cells 36, 37. Conversely, the NEC displayed one or two large vacuole(s), a small nucleo‐cytoplasmic ratio, electron lucent cristae, and small endoplasmic reticulum profiles (Fig. 1B,D,F, and F’), features coupling the cell division to the cell expansion growth 37.

The expression profiles of the cyclin genes, *VvCYCD3;3* and *VvCYCA2;2,* also demonstrated the differences in cell division activity of EC and NEC (Fig. 2C,D,E, and F). The choice of these two distinct types of cyclins shed light on two crucial transitions between the phases of cell cycle, the G1/S and the G2/M, respectively 38. The *VvCYCD3;3* gene belongs to the D‐type cyclins known to display a certain functional redundancy required for normal embryonic development 39. The expression of *CYCD3* genes has been demonstrated as regulated at transcriptional level by cytokinins, and this hormonal control is efficiently mimicked by sucrose, which also acts as a metabolic signal 40, 41. Thus, the presence of cytokinins and sucrose in fresh culture medium may explain the sharp induction of *VvCYCD3;3* after the subculture of NEC. Inversely, the decrease in cyclin gene expression at the stationary phase due to the sucrose depletion argues in favor of a strong but anarchic proliferation of NEC. As sucrose availability is crucial for commitment to plant cell division during G1 phase by controlling the expression of D‐type cyclins 42, a plausible explanation for the strong expression of *VvCYCD3;3* in EC may consist in their partial synchronization in G1 phase due to the sucrose starvation. The sustained expression of both cyclin genes provides evidence for a moderate but organized proliferation of the EC throughout the subculture period, assumption supported by the specific profiles of expression of many cell‐cycle genes in synchronized cell cultures of Arabidopsis 43, 44.

In Arabidopsis seeds during the embryo development, the enhanced cell proliferation has been associated with the increased expression of D3‐ and B1‐type cyclins in *megaintegumental/auxin response factor 2* (*mnt/arf2*) and *apetala 2* (*ap2*) mutants, affected in the corresponding transcription factors responsible for the downregulation of these cyclins 45, 46. The triple *Arabidopsis* mutant *cycd3;1/2/3* is characterized by a reduction in cell number compensated by an increase in cell size and DNA content 47. Furthermore, it has been demonstrated that transcripts of *CYCA* genes, such as *CYCA2;1* and *CYCA2;3,* accumulate at the G2 to M transition in a manner concomitant with these of all *CYCB* genes. The fact that *CYCA2;1* expression is related to cell division was further confirmed by the promotion of the endoreduplication by its proper mutation 48.

### Metabolic behavior and profiling of glycolysis enzyme activities

**Figure 9:**
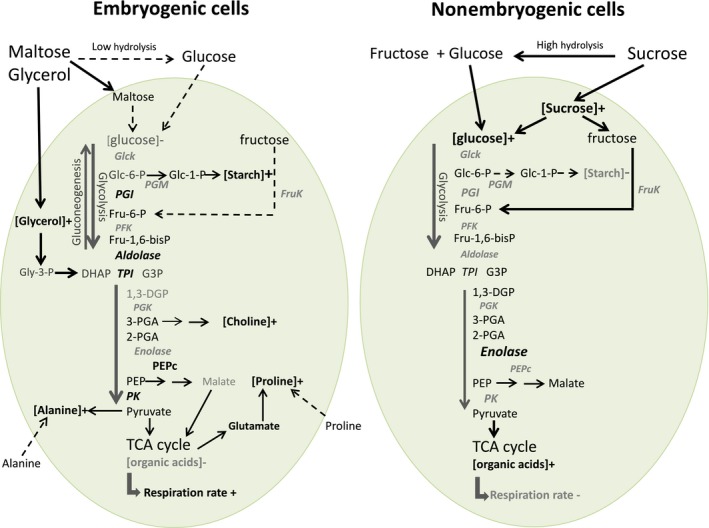
Schematic presentation summarizing the main differences in glycolysis pathway efficiency of grape EC and NEC. Enzyme abbreviations: GK—glucokinase, FK—fructokinase, PGM—phosphoglucomutase, PGI—phosphoglucose isomerase, PFK—phosphofructokinase, TPI—triose‐phosphate isomerase, PGK—phosphoglycerokinase, PEPC—phosphoenolpyruvate carboxylase, PK—pyruvate kinase. The names and the arrows in bold correspond to higher enzyme activities and metabolite fluxes than those in normal font and dashed lines.

The detailed ^1^H‐NMR analysis of intracellular metabolites emphasized some marked metabolic discrepancies between EC and NEC summarized in Fig. 9. It appears that EC are able to maintain a low intracellular content of glucose and sucrose, in parallel with the synthesis and storage of starch, amino acids (in particular proline and alanine), choline, and proteins. Considering the fact that EC have a relatively low but maintained division activity, the influx of maltose coupled with a less efficient glycolysis (glycerol effect) may explain the starch accumulation in numerous amyloplasts. Inversely, NEC demonstrated a high intracellular level of glucose and sucrose, as well as of some TCA cycle intermediates (citrate, succinate, fumarate, malate).

Although sucrose is the classical sugar in cell suspensions, the use of maltose in embryogenic cell culture and the subsequent induction of somatic embryogenesis are now largely applied for numerous plant species 36, 49, 50, 51, 52. The most credible reason for understanding the beneficial effect of maltose on somatic embryogenesis consists in the low hydrolysis of this disaccharide by the extracellular α‐glucosidase, which has been at least five times slower than the sucrose hydrolysis by the cell wall invertases in barley microspore culture 53. The assimilation of supplied carbon from [^14^C]sucrose has been demonstrated to be remarkably greater than that from [^14^C]maltose. In addition, the authors provided evidence that the adenylate energy charge (ATP and ADP) increased, when the microspores were transferred to maltose, and, inversely, it decreased after transfer to sucrose at the same concentration (40 mM).

Contrary to most yeast species relying on glycerol as a carbon substrate in aerobic conditions, plant cells do not use it efficiently as a growth substrate. For example, in mesophyll cells from barley leaves, glycerol triggers an accumulation of glycerol‐3‐phosphate at the expense of intracellular Pi and impairs phosphate metabolism and photosynthesis 54. The growth of excised maize root tips is also inhibited in the presence of 100 mM glycerol 55. Glycerol, which may provide carbon to the glycolysis via a phosphorylation (glycerokinase) followed by an oxidation (glycerol‐3‐phosphate dehydrogenase), is not only a poor substrate for plant cell growth, but also a disturbing factor, which slows down the glycolysis flux. This is consistent with the known inhibition of glucose‐6‐phosphate isomerase by glycerol‐3‐phosphate 56. Two independent analyses realized on citrus and chicory cells cultured on medium containing glycerol as only carbon source have demonstrated the synthesis of soluble sugars (sucrose, glucose, fructose) as well as of starch 57, 58. These latter observations are in agreement with the starch accumulation in grape EC and argue in favor of a possible role of glycerol in the stimulation of gluconeogenesis.

It is very likely that NEC strongly rely on fermentative metabolism to generate ATP, as fermentation products ethanol and formic acid were found in the culture medium (Fig. 6), and enolase activity was very high compared to EC (Fig. 8). It is striking that high enolase characterizes a range of cancer cells but also yeast 59, which both use the Warburg effect to increase their cell proliferation rate under favorable conditions (for instance high sugar supply). The partial switch of NEC to fermentation was corroborated by their more rapid cell growth (Fig. 2A,B) and confirmed by their two times lower oxygen consumption rate (Fig. 7) than those of the EC. Moreover, due to low energy efficiency, fermentative metabolism requires a high metabolic flux through glycolysis. Inversely, phosphoenolpyruvate carboxylase and pyruvate kinase activities were much higher in EC compared to NEC (Fig. 8). One explanation could be that in EC, these activities need to be relatively high to support the supply of carbon into the TCA cycle, and thus, the synthesis of ATP and carbon skeletons to cell growth.

### Cross‐talk of hormonal and metabolic regulation

In cells displaying embryogenic potential but maintained on noninducing medium, auxin depletion induces the synthesis of α‐amylases and the stimulation of starch catabolism 60. The same authors have demonstrated the importance of metabolic regulation, namely the fact that the decrease in soluble sugars level may produce the same derepression effect on the genes encoding starch degradation enzymes. The already‐described opposite effects of auxins and cytokinins on amyloplast development and starch synthesis genes in the BY2 tobacco cells 61, coupled to the quick sucrose metabolism fueling the high rate of cell division, are therefore arguments in favor of the low starch content in NEC. Inversely, the EC are cultured at the conditions of high auxin repression of starch degradation. Their ability to maintain a low cellular level of glucose may be due to the maltose and the glycerol as carbon sources, which impose a low glycolysis flux at the expense of starch accumulation. Our model is a pertinent example that the low glycolysis flux is required to prevent the EC from differentiating. The low glycolytic metabolism under high auxin pressure thereby hinders the differentiation of the EC through somatic embryogenesis.

## Conclusion

Our results fill in the literature gap by providing evidence for the marked metabolic differences between EC and NEC. These new data highlight the NEC as a model of strong and anarchic cell proliferation, while the EC as a model of cell ability to switch from moderate and organized cell proliferation toward differentiation. These both cell types with differential efficiency of glycolytic metabolism and distinct cell fate open perspectives for the study of gene expression regulation in the glucose signaling pathways, at the cross‐talk of hormonal and metabolic signals.

## Author contributions

RA, JP, DR, YG, and PF designed the experiments. JP, CG, JV, MM, RAA, and FT performed the experiments. RA, JP, DR, YG, and PF discussed the data. RA wrote the manuscript. RA, DR, and YG revised the article. All authors approved the final manuscript.

## Funding

This work was supported by the National Center of Scientific Research, the University of Poitiers, the Poitou‐Charentes Region (PhD grant of JP), the ERASMUS student program (Master's degree grant to RAA), the Metabolome Platform of Functional Genomics Center of Bordeaux‐MetaboHUB, the National Institute of Agricultural Research Bordeaux‐Center, the State‐Region Planning Contracts (CPER), and the European Regional Development Funds (FEDER).

## Conflict of interest

The authors declare that the research was conducted in the absence of any commercial or financial relationships that could be considered as a potential conflict of interest.

## Supporting information


**Fig. S1.** ^1^H‐NMR spectra of cellular metabolites. EC (red), NEC (black).
**Fig. S2.** ^1^H‐NMR spectra of metabolites in culture media. EC (red), NEC (black).
