# Supplementary figures and images for: Comparative metabolomics and glycolysis enzyme profiling of embryogenic and nonembryogenic grape cells
Source: FEBS Open Bio. 2018 Apr 17;8(5):784–98. doi: 10.1002/2211-5463.12415 (PMC5929931; doi:10.1002/2211-5463.12415)

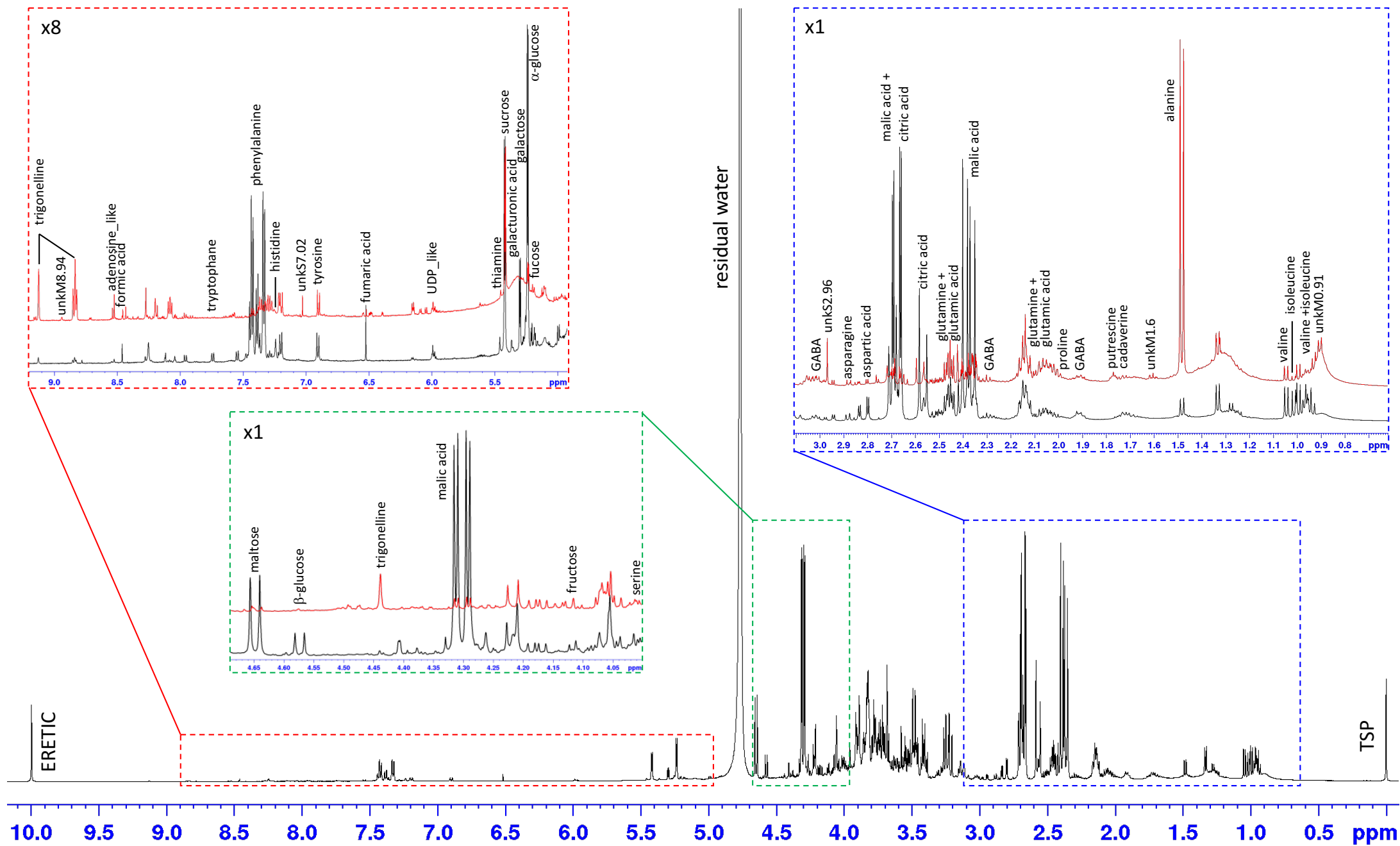

Figure S1

Figure S2

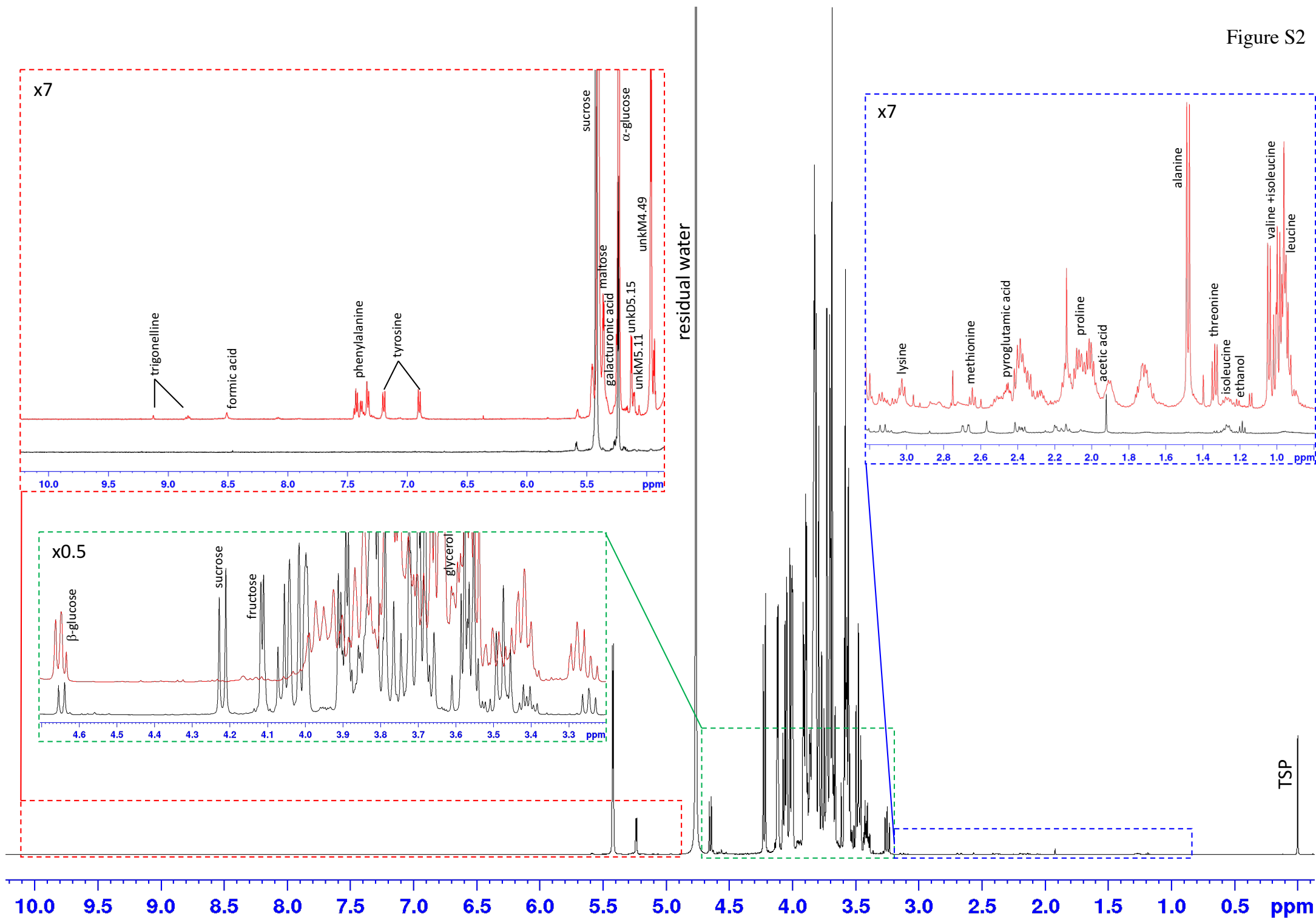

Supplement: Supplementary file 1 — Fig. S1. 1H‐NMR spectra of cellular metabolites. EC (red), NEC (black). Fig. S2. 1H‐NMR spectra of metabolites in culture media. EC (red), NEC (black). [file FEB4-8-784-s001.pdf]
